# Machine Learning Enables Prediction of Halide Perovskites’ Optical Behavior with >90% Accuracy

**DOI:** 10.1021/acsenergylett.2c02555

**Published:** 2023-03-10

**Authors:** Meghna Srivastava, Abigail R. Hering, Yu An, Juan-Pablo Correa-Baena, Marina S. Leite

**Affiliations:** † Department of Materials Science and Engineering, UC Davis, Davis, California 95616, United States; ‡ Department of Materials Science and Engineering, Georgia Institute of Technology, Atlanta, Georgia 30332, United States

## Abstract

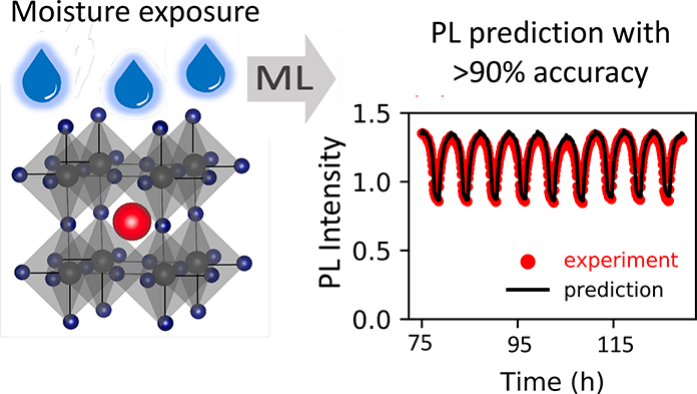

The composition-dependent degradation of hybrid organic–inorganic perovskites (HOIPs) due to environmental stressors still precludes their commercialization. It is very difficult to quantify their behavior upon exposure to each stressor by exclusively using trial-and-error methods due to the high-dimensional parameter space involved. We implement machine learning (ML) models using high-throughput, *in situ* photoluminescence (PL) to predict the response of Cs_*y*_FA_1–*y*_Pb(Br_*x*_I_1–*x*_)_3_ while exposed to relative humidity cycles. We quantitatively compare three ML models while generating forecasts of environment-dependent PL responses: linear regression, echo state network, and seasonal autoregressive integrated moving average with exogenous regressor algorithms. We achieve accuracy of >90% for the latter, while tracking PL changes over a 50 h window. Samples with 17% of Cs content consistently showed a PL increase as a function of cycle. Our precise time-series forecasts can be extended to other HOIP families, illustrating the potential of data-centric approaches to accelerate material development for clean-energy devices.

Hybrid organic–inorganic perovskites (HOIPs) are a promising class of material for the development of energy-efficient devices ranging from solar cells to light-emitting diodes (LEDs).^1−3^ In HOIPs, with an ABX_3_ structure, the A-site commonly contains organic cations such as methylammonium (MA^+^) and formamidinium (FA^+^), inorganic cations such as Cs^+^ or Rb^+^, or a mixture. The B-site is almost exclusively occupied by Pb^2+^, though some work has explored lead-free alternatives using Sn.^4^ Halides—typically I^–^, Br^–^, or Cl^–^, or some mixture of the three—occupy the X-site. Compositional tuning of the A- and X-sites enables tailoring of the bandgap, an essential property for optoelectronic applications. As these materials approach commercialization, several experimental challenges slow their advancement, including the vast compositional parameter space available and the complex, sometimes convoluted, contribution of environmental stressors to their still limited stability.^5,6^ Material degradation occurs through different processes, including decomposition, degassing, phase transitions, and phase segregation, depending on the chemical composition and the set of environmental stressors applied.^7,8^

Environmental conditions, such as moisture, affect the optical behavior (e.g., photoluminescence’s peak location, value, and full-width half-maximum)^9−13^ of HOIPs, with material changes that are dynamic over time and often nonlinear.^14^ They are also heavily composition-dependent, making comparison difficult even between perovskites from the same family.^15,16^ Traversing the large HOIP compositional parameter space and quantifying the effect of all stressors (and their combinations) is unfeasible on the time scale needed to commercialize and meet net zero carbon emissions goals. Yet, machine learning (ML) can accelerate the discovery of stable HOIPs by compositional screening^17,18^ by automated and autonomous synthesis/characterization,^19,20^ and by learning trends between compositional ratios and responses to environmental stressors.^5,6^ Further, time-series predictions tracking photoluminescence (PL) over changing environmental conditions can simulate real-world operating conditions and provide an estimation of how HOIP solar cells will perform in the future, akin to a weather forecast.

Here, we demonstrate how ML can be implemented to track changes in radiative recombination of selected Cs_*y*_FA_1–*y*_Pb(Br_*x*_I_1–*x*_)_3_ thin films, under repeated 6 h rH cycles that mimic accelerated day-night weather variations based on typical summer days in northern California. Using a high-throughput setup, we obtain 50 PL spectra every hour and 7200 spectra over the course of a single experiment, sufficient for a robust ML-driven analysis. For a single cycle, all compositions interrogated display a PL enhancement with increasing rH as H_2_O passivates bandgap trap states and suppresses nonradiative recombination. Surprisingly, FA-rich films show the greatest PL increases over the course of the rH cycling, while Cs-rich films reach a plateau in maximum PL value after 5–10 cycles. The rH-cumulative features presented in the PL responses are chemical-composition-dependent, justifying the need for applying ML methods that are composition-agnostic. We apply three ML models to the data sets and generate forecasts of environment-dependent PL responses and quantitatively compare their accuracy. We use linear regression (LR), echo state network (ESN), and seasonal auto-regressive integrated moving average with exogenous regressors (SARIMAX) algorithms and find average normalized root-mean-square error (NRMSE) values of 54, 47, and 8%, respectively. The very high and consistent accuracy of SARIMAX, even when tracking long-term changes over a 50 h window, showcases this algorithm’s capability to model complex, nonlinear data from varied HOIP compositions. Overall, the precise time-series forecasts illustrate the potential of data-centric approaches for HOIP stability investigations and stages the promise of automation, data science, and ML as tools to drive the further development of this emerging material.

We choose the archetype Cs_*y*_FA_1–*y*_Pb(Br_*x*_I_1–*x*_)_3_ family and the Cs-FA and Br-I compositional spaces considering their bandgap variability and potential applications in photovoltaics. To avoid the detrimental effects of MA^+^, the A-site is instead occupied by formamidinium (FA^+^) and cesium (Cs^+^). Table 1 displays the specific chemical compositions used in our environmental PL cycles. The selected samples represent well the variability of material response upon exposure to moisture, ultimately constituting a model system for the ML-based analyses presented here. All *in situ* experiments are performed simultaneously (see Figure S1 for details regarding our automated setup), which assures that *all* samples are submitted to the very same environmental stressor conditions.

**Table 1:** Compositional Space of the Perovskite Films^a^

Composition Cs_*x*_FA_1–*x*_Pb(I_*y*_Br_1–*y*_)_3_	Cs (%)/Br (%)
Cs_3/6_FA_3/6_PbI_3_	Cs-50%/Br-0%
Cs_3/6_FA_3/6_PbBr_1/2_I_5/2_	Cs-50%/Br-17%
Cs_3/6_FA_3/6_PbBr_1_I_2_	Cs-50%/Br-33%
Cs_2/6_FA_4/6_PbBr_1/2_I_5/2_	Cs-33%/Br-17%
Cs_1/6_FA_5/6_PbBr_1/2_I_5/2_	Cs-17%/Br-17%

a Ratios of Br:I and Cs:FA are varied simultaneously.

To quantify and deconvolute the effects of rH on the HOIP samples, we first track the transitions in PL over the course of one rH cycle. The initial increase to 70% rH produces a significant enhancement in radiative recombination, see Figure 1a–e. As expected, as the rH decreases to <5% (Figure 1f–j), a corresponding decrease in PL peak value is observed. Similar effects have been reported in the literature.^21,22^ The presence of moisture in the environment passivates trap states located within the semiconductor bandgap. Consequently, there is a reduction in nonradiative recombination events, giving rise to this seemingly counterintuitive behavior. The initial PL spectra before any rH exposure are shown in Figure S2. Note that, as desired, the PL signal is unchanged during the time-dependent tests at low rH, see Figure 1k–o. It is important to deconvolute any potential effects of time-dependence from the influence of rH onto material degradation.

**Figure 1:**
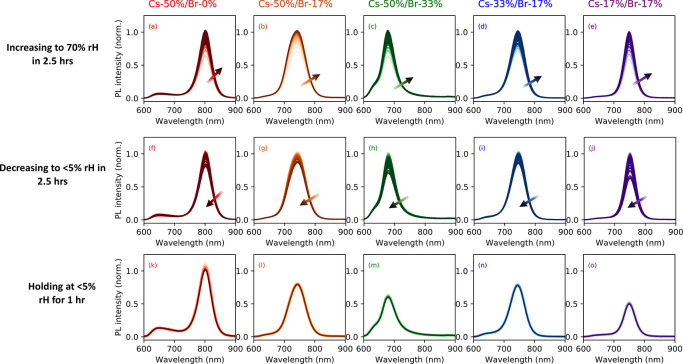
Spectral evolution of PL emission during humidity cycle. PL spectra as the relative humidity (rH) is increased to 70% within 2.5 h, decreased to <5% over 2.5 h, and then held at <5% for 1 h for samples (a–c) Cs-50%/Br-0%, (d–f) Cs-50%/Br-17%, (g–i) Cs-50%/Br-33%, (j–l) Cs-33%/Br-17%, and (m–o) Cs-17%/Br-17%. Arrows denote passage of time as the spectrum color changes in hue darkness. Spectra for all samples are collected every 6 min.

To visualize the long-term spectral evolution from rH cycling, we plot a series of spectra acquired under identical environmental conditions (rH < 5%, *T* = 22 °C) for each sample (Figure 2a–e), after the 2nd, 7th, 12th, and 17th cycles. The final, darkest spectrum was taken after all 18 rH cycles were completed and the samples were held in an inert rH < 5% condition for 10 h. These final spectra are to verify that the samples recovered from any transient, water-induced chemical processes, as desired for our ML-based analysis. Notably, the pure-I composition (Cs-50%/Br-0%, in Figure 2a) shows negligible peak shifting despite a significant decrease in overall PL intensity. Because I^–^ is larger than Br^–^, it distorts the atomic lattice of the perovskite, weakening atomic orbital overlap and reducing the bandgap. Ion migration in mixed-halide compositions into I^–^ rich and Br-rich domains will, thus, manifest macroscopically as a decrease in bandgap, i.e., a shift to longer PL wavelengths accompanied by a broadening of the emission peak.^23,24^ Concurrently, samples Cs-50%/Br-17% and Cs-33%/Br-17% (Figure 2b,d, respectively) exhibit a minor (∼10 nm) red shift in PL peak location and an overall broadening of the PL emission spectrum over repeated rH cycling. Therefore, we attribute the observed multicycle trends in peak location and width to halide segregation, as previously observed in this perovskite family.^25,26^

**Figure 2:**
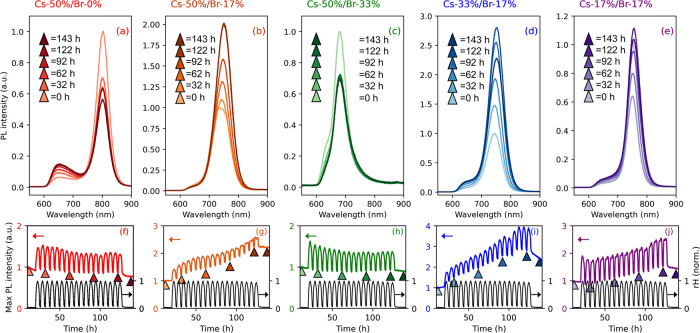
Effect of humidity cycling on PL. Top row: PL spectra for samples (a) Cs-50%/Br-0%, (b) Cs-50%/Br-17%, (c) Cs-50%/Br-33%, (d) Cs-33%/Br-17%, and (e) Cs-17%/Br-17% at five time points during the 118 h experiment (corresponding to 18 rH cycles). Each spectrum is acquired under identical environmental conditions (22 °C, rH < 5%) after 0, 32, 62, 92, 122, and 143 h. Bottom row: Maximum PL intensity for samples (a) Cs-50%/Br-0%, (b) Cs-50%/Br-17%, (c) Cs-50%/Br-33%, (d) Cs-33%/Br-17%, and (e) Cs-17%/Br-17% subjected to relative humidity (rH) cycling for 108 h (total experiment time is 144 h). The normalized rH profile is shown (black line) on each plot, see right *y*-axis. The left *y*-axis denotes the normalized PL. Each 6 h cycle ranges from <5 to 70% rH. The color-coded arrows correspond to the selected spectra shown in (a–e).

In terms of the peak PL value, the A-site composition exerts significant control over the multicycle behavior. Compositions with a 1:1 Cs:FA ratio (Figure 2a–c) display contrasting effects, some exhibiting a decrease in PL intensity (Cs-50%/Br-0% and Cs-50%/Br-33%) and one showing a significant increase (Cs-50%/Br-17%). These opposing trends could be due to local phase segregation, microstructural inhomogeneity and voids, or structural distortions dependent on the Br:I ratio.^27,24^ The Cs-poor compositions (Cs-33%/Br-17% and Cs-17%/Br-17% in Figure 2d,e, respectively) display the greatest moisture-induced enhancement in radiative recombination. Overall, these measurements demonstrate the remarkably complex interplay between rH and PL in perovskite films, which we show is heavily dependent on both composition *and* time of exposure.

While the general trend of humidity-induced PL enhancement is consistent for all samples, we observe composition-dependent fluctuations in behavior both across single cycles and over the course of several days of cycling, as seen in Figure 2f–j. Here, we track the maximum PL intensity as a function of rH, as this is an ideal input to train, validate, and test the ML models, as will be discussed later. The high-Cs^+^ perovskites show drastically different behavior. Rather than accumulating PL enhancement over time, the material in both Cs-50%/Br-0% and Cs-50%/Br-33% (Figure 2f,h) undergoes a decrease and subsequent plateau after 5–10 rH cycles. Once this plateau is reached, the accumulated PL enhancement after each rH cycle is minimal. These samples have intermediate Cs:FA ratios and are located at the extremes in terms of tested halide content. Interestingly, the intermediate halide composition (Cs-50%/Br-17%, shown in Figure 2g) does not display the same trend. Here, the PL signal increases almost monotonically with cycles. Conversely, the lowest Cs^+^ content sample (Cs-17%/Br-17%, Figure 2j) presents high sensitivity to the presence of moisture, where we detect consistent variations in PL peak intensity for nearly all cycles. Further investigation is needed to determine the specific physical mechanisms for PL enhancement and decay in these samples, an important study that is beyond the scope of this work, as we choose to focus on the accuracy of ML models instead. Note that the nonlinear optical response of this perovskite family makes it an ideal model system to implement and assess ML routines.

The large amount of data acquired in our experiments, >7000 PL spectra within 6 days, is sufficient to train predictive ML models without the need for data augmentation. Thus, PL is an ideal method for quantitatively comparing distinct ML models. We apply three ML algorithms of varying complexity to time-series PL data: linear regression (LR), echo state network (ESN), and seasonal autoregressive integrated moving average with exogenous factors (SARIMAX). Here, we focus solely on computational methods to identify the most promising approach for HOIP time-series forecasting while splitting the experiment into a train/validation set and a test set, see Figure 3a. To evaluate how well these ML models can predict over a compositional range, we use standardized methods across the sample set. The three algorithms are independently trained and tested on each of the sample compositions. This adaptability is critical given the vast compositional space for HOIPs. The practicality and time effectiveness of ML in the context exploited here would be severely reduced if different optimization processes requiring human inputs were necessary to train a different model for every sample (in this case, the human time cost to fit all samples would be increased 10×). See the SI file for a detailed description of the data acquisition and analysis methods.

**Figure 3:**
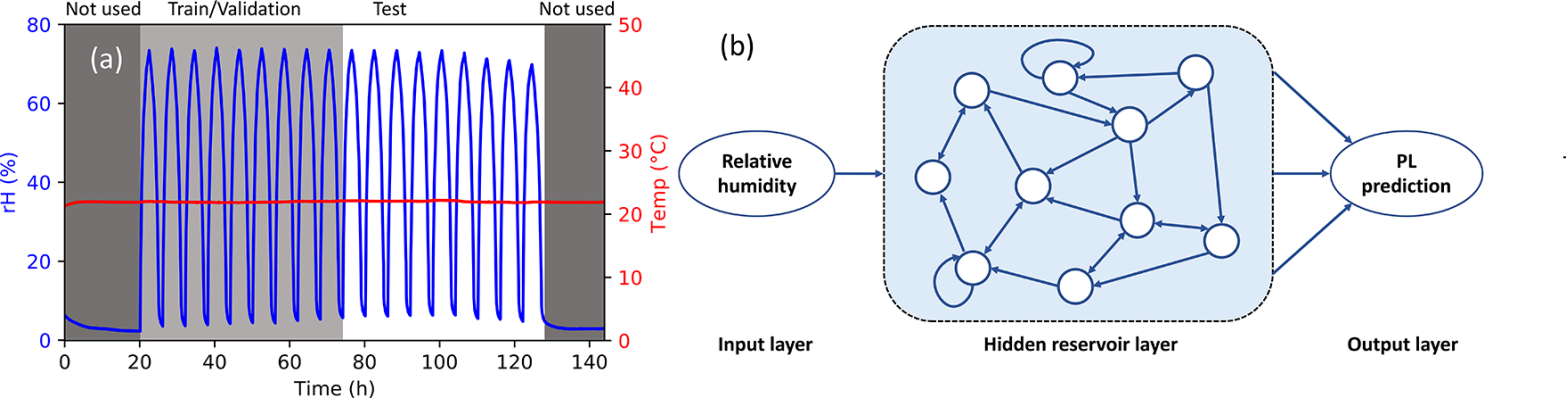
Machine learning models to forecast PL response in halide perovskites. (a) Relative humidity (rH) cycling data is split into training/validation and testing sets. (b) Schematic of echo state network (ESN) to forecast environmental PL. Variable rH inputs enter a sparsely connected reservoir of neurons, which outputs a prediction for the PL value. During training, the network updates the input, reservoir, and output weight matrices to minimize error between its PL predictions and the experimental data. At the testing stage, the weights are constant, and the ESN generates forecasts based only on the rH data at each time point.

First, we implement a baseline LR algorithm using a 50%–50% train-test split. The model uses rH and PL training data to determine the regression coefficients. For the test set, rH data points (Figure 3a) are inputted to the model, which then generates a PL forecast. The test results are displayed in Figure 4a–e for all samples, where the black lines are the regression predictions, and the colored circles are the experimental data. The predictive performance is highly variable between samples, and the normalized root-mean-square error (NRMSE) values range from ∼92% (Cs-33%/Br-17%) to 23% (Cs-50%/Br-0%). However, the average across all samples is 54%, with an overall poor visual fit. Note that although a separate regression is generated for each sample, the process is completely automated, and the fitting takes <0.01 s per sample on a computer with 16 GB of RAM. Therefore, this is a composition-agnostic approach with very high computational efficiency.

**Figure 4:**
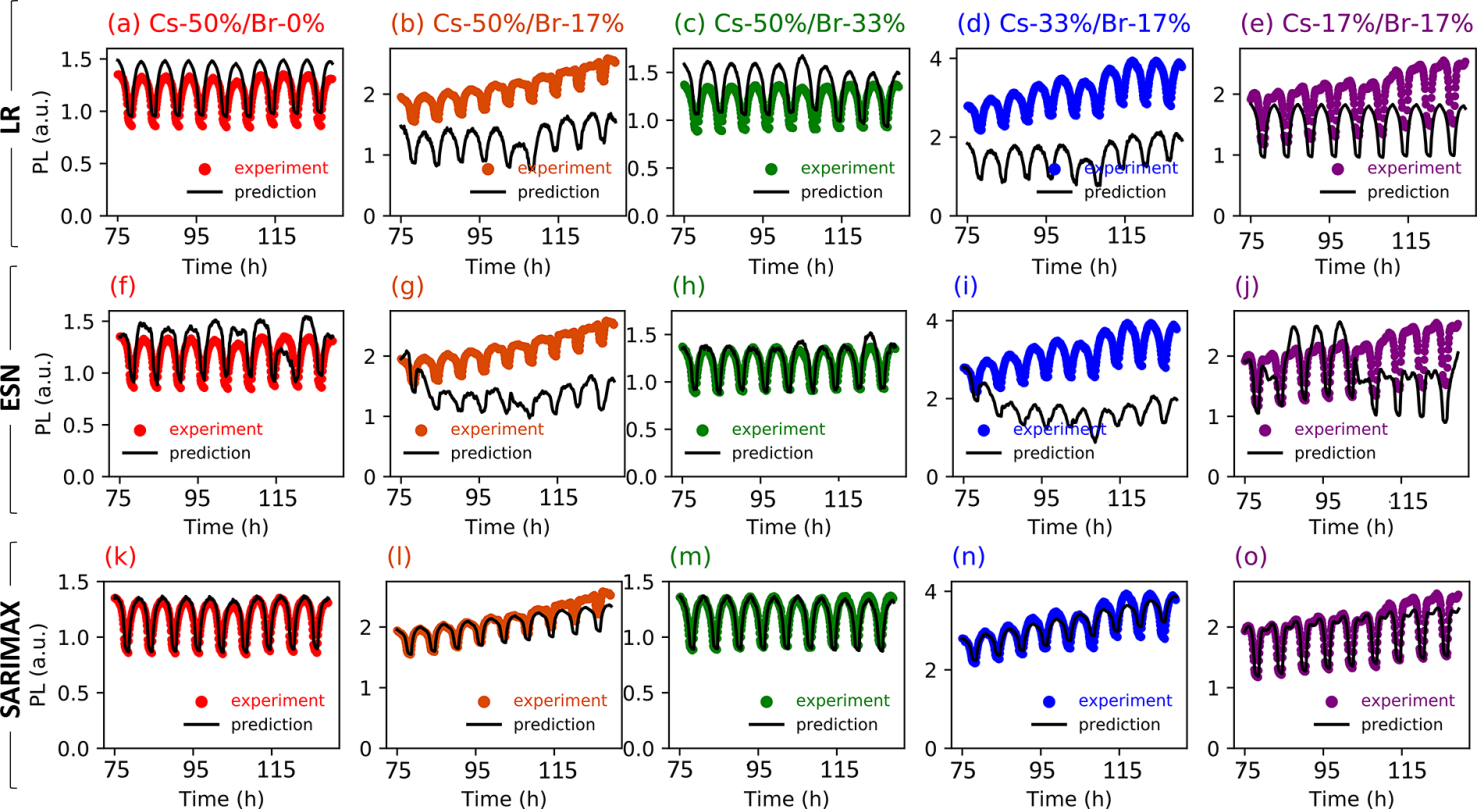
Forecasting humidity-dependent PL. Test set predictions (black lines) and seasonal experimental data (colored dots) for linear regression (LR), echo state network (ESN), and seasonal autoregressive integrated moving average with exogenous regressors (SARIMAX) for (a,f,k) Cs-50%/Br-0%, (b,g,l) Cs-50%/Br-17%, (c,h,m) Cs-50%/Br-33%, (d,i,n) Cs-33%/Br-17%, and (e,j,o) Cs-17%/Br-17%. In all cases, relative humidity (rH) is the sole input to the models during testing.

To improve upon the LR prediction accuracy while preserving computation efficiency, we develop an ESN to model the data, a form of recurrent neural network (RNN) where “recurrent” means that the network retains a dynamic memory of past states, see Figure 3b.^28^ The ESN performs modestly better than the LR model, as displayed in Figure 4f–j. Here, we subdivide the nontest data into training and validation sets, with a 25%–25%–50% train-validation-test split, where past PL values and trends are used to generate predictions. For all compositions, we implement a 250-node ESN with a sparsity of 0.1 (meaning 10% of recurrent weights are set to zero). The hyperparameter optimization is shown in Figure S3 (see the SI for further details.) These values are selected to provide a balance between complexity and computational efficiency.^29^

The ESN model still diverges for a few samples, requiring us to use a more sophisticated algorithm: seasonal autoregressive integrated moving average with exogenous regressors (SARIMAX). This statistical modeling approach sets interpretable coefficients during the fitting process, like LR, an advantage over black box neural networks like ESN. Our SARIMAX entails a 50%–50% train-test split, two differencing steps and two moving average terms which use observations at previous time steps (see SI for details). The accuracy enhancement is dramatic in this case: the PL response of all samples can be predicted with high exactitude, regardless of global and local trends. SARIMAX, like all other algorithms presented here, uses a composition-agnostic approach so that human input is only required once.

Concerning the details regarding SARIMAX, seven interpretable coefficients are selected, represented by variables (*p,d,q*)(*P,D,Q,s*), as described in the SI file. The integrative parameter (*d*) corresponds to the order of differencing, where a differenced time series is equal to the change between points in the original time series. We apply one order of differencing (*d* = 0 or 1) and one order of seasonal differencing (*D* = 1). These mathematical operations are needed to produce a stationary time series—that is, a series in which the statistical properties (such as mean and variance) do not vary over time—which is a prerequisite for statistical modeling. Autoregressive terms (*p*) are then added to incorporate the effects of past PL measurements on the PL at the current time. For example, *p* = 1 adds a term for the PL value at *t* – 1. No autoregressive terms are included as the effect of previous data points is negligible after differencing (Figures S4 and S5). Moving average terms (*q*) track the change in the series over time by averaging consecutive terms. We apply one moving average term (*q* = 1) and one seasonal moving average term (*Q* = 1). We also set the seasonality parameter (*s*) to 60, the number of data points contained in each day-night cycle, for a final SARIMAX with coefficients (0,1,1)(0,1,1,60). The exogenous variables, rH and temperature, serve as additional inputs to the model.

A model performance comparison for the rH cycling prediction task is shown in Figure 5. We use the normalized root-mean-square error (NRMSE) metric, which is scale-invariant and enables direct comparison between ML models. As expected, the LR model has the highest NRMSE for nearly every sample, followed by the ESN, and finally the SARIMAX. The rH cycling created nonlinear responses in the perovskite films (see Figure 4), likely due to the complexity of the physical mechanisms involved, including water adsorption and trap state passivation. The patterns in the time series for the rH cycling caused low prediction accuracy for the LR and the ESN, with average NRMSEs of 54 and 46%, respectively. By adding seasonality trends and moving average terms to a neural network, we establish an SARIMAX model with an average prediction NRMSE of only 8% over the 54 h prediction window (eight day-night cycles). The highest NRMSE found, equal to 13%, is accounted for Cs-50%/Br-17% because after 30 h of prediction the SARIMAX function slightly loses track of the past trends. Additional valuable information could be interpreted from the PL spectra, such as the full-width half-maximum (fwhm) of the main peaks and the integrated PL signal (the full area under the PL spectra) (see Figure S6). We use this information to further train the SARIMAX models, complementing the data gathered from the PL intensity trends, which do not “tell the full story” of how the material is changing upon exposure to rH. We note that the average NRMSE is higher than that of the PL peak intensity in our case, as the fwhm and integrated PL have more irregular responses to the cyclical rH inputs. This result indicates that further refinement of the SARIMAX algorithm is needed to fully describe and predict PL spectra, including wavelength-dependent changes, which is beyond the scope of this work. SARIMAX models could also be combined with another recurrent neural network, such as long short-term memory,^5^ to predict the optical response of chemical compositions beyond the ones trained, maintaining the time-correlation originally learned by the model while discovering composition-dependent trends. Overall, the extremely high precision for the SARIMAX model is offset by its computational cost and fitting time, which is 1–2 orders of magnitude longer than that of the ESN. We quantify the consequences of this trade-off by comparing the SARIMAX fitting time to the length of the prediction window. We calculate a 1:1500 ratio between these times, indicating that the fitting process remains highly effective for this task.

**Figure 5:**
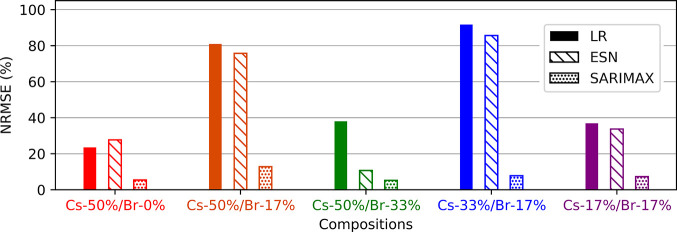
Quantitative comparison of ML algorithms. Normalized root-mean-square error (NRMSE) values of PL forecasts produced by LR (solid), ESN (dashed), and SARIMAX (dots) models.

In conclusion, we demonstrated the capabilities of ML to forecast the PL environmental response in a series of Cs_*y*_FA_1–*y*_Pb(Br_*x*_ I_1–*x*_)_3_ perovskites. Using rH cycling data, we trained three algorithms, LR, ESN, and SARIMAX, and quantitatively compared their accuracy. We found that seasonal neural networks, such as SARIMAX, can predict material behavior with >90% accuracy during an eight day-night rH cycle. For a quantitative comparison between three ML models, we first sorted and preprocessed the data, combining rH and PL observations into time-correlated “data points.” We then trained and validated the models on the first 50% of each data set and used the remaining 50% to test their ability to predict the maximum PL value at every time step with only the rH as input. The models obtained average errors of 54% (LR), 47% (ESN), and 8% (SARIMAX) over 50+ hours. For every ML task, we ensured that our methods were composition-agnostic, meaning that the same process could be used for other types of HOIPs. Our results showed that LR is not an adequate approach for nonstationary time series. Yet, the use of neural networks to forecast the PL response is very suitable for analyzing HOIPs’ changes upon exposure to rH. The generalizability of our methods to multiple compositions can help shorten the time required for compositional tuning, which is currently a major bottleneck in the design process of HOIP for light-absorbing and -emitting devices. Specifically, the combination of SARIMAX with long short-term memory (LSTM) models could enable the prediction of perovskite chemical compositions beyond the training set, which, in turn, would lead to an accurate estimate of the stability of currently underexplored compositions. We envision extensions of this work to include other environmental stressors beyond moisture (such as oxygen, temperature, light, and bias). Combinations of many stressors could mimic operating conditions in various geographic locations, providing insight into HOIP solar cells’ stability without necessitating lengthy experiments at each individual location.

## Supporting Information Available

Sample fabrication details, automated setup for environmental PL, relative humidity control, and machine learning models (PDF)
