# Supplementary material for: Machine Learning Enables Prediction of Halide Perovskites’ Optical Behavior with >90% Accuracy
Source: ACS Energy Lett. 2023 Mar 10;8(4):1716–22. doi: 10.1021/acsenergylett.2c02555 (PMC10112389; doi:10.1021/acsenergylett.2c02555)
Supplement: Supplementary file 1 — nz2c02555_si_001.pdf [file nz2c02555_si_001.pdf]

Supporting Information

Machine Learning Enables Prediction of Halide Perovskites' Optical Behavior with >90% accuracy

Meghna Srivastava[1#], Abigail R. Hering[1#], Yu An[2], Juan-Pablo Correa-Baena[2], and Marina S. Leite[1*]

[1]Department of Materials Science and Engineering, UC Davis, Davis, CA, 95616, USA
[2]Department of Materials Science and Engineering, Georgia Institute of Technology, Atlanta, GA, 30332, USA

*Corresponding author: mleite@ucdavis.edu

I. Sample fabrication

The samples are fabricated following the procedure described in ref. [1] The perovskite precursor solution is spin-coated onto a conductive glass, fluorine-tin-oxide (FTO) coated substrate pre-heated to 65◦C using a two-step spin-coating process. The first spin-coating step is 10 seconds at 500 rpm with an acceleration of 250 rpm s-1. An antisolvent, CB (250 μL) is dripped onto the sample 5 seconds before the end of the second step. Following the spin-coating, all films are annealed for 5 minutes at 65°C. Due to the poor solubility of CsBr in common solvents like DMF and DMSO, 0.4 M perovskite precursor solutions are prepared for thin film fabrication. Ratios of Cs:FA and Br:I are tuned using four master solutions of 0.4 M $CsPbI_3$, $FAPbI_3$, $CsPbBr_3$, and $FAPbBr_3$. The master solutions are generated by dissolving combinations of chemical precursors under mild heat at 65◦C for 1 hour. Solutions are prepared in a N_2-filled glovebox with <2 ppm of O_2 and H_2O. Perovskite solutions with desired $Cs_yFA_{1-y}Pb(Br_xI_{1-x})_3$ compositions are produced by mixing the master solutions in corresponding molar ratios.

II. Automated setup for environmental PL

To acquire sufficient data to train complex ML models such as deep neural networks, we use photoluminescence (PL), a high-throughput sample characterization method. Because perovskite thin film degradation occurs on a scale of days, it is extremely useful to measure multiple samples over the course of a single experiment. Typical bulk PL set-ups do not have this capability, consisting of a small, stationary sample chamber that is exposed to laser illumination either continuously or at fixed intervals while data is acquired.[2] Here, we introduce a characterization system enabling efficient data collection from many samples at once, see Figure S1. The design objectives for the new system are:

(i) Acquires data from several samples during a single experiment

(ii) Exposes all samples to the same set of environmental stressors such that data can be directly compared

(iii) Allows for automated data analysis and real-time monitoring

(iv) Ensures reproducibility and consistent conditions throughout experiments lasting up to seven days

The sample chamber is mounted onto a custom-built translation stage consisting of two linear actuators with NEMA 23 stepper motors. Both actuators are fitted with flat platforms to mount samples or other structures. We mount one actuator directly to the optical table, referred to as the "x-direction actuator." The second actuator, referred to as the "y-direction actuator," is mounted vertically onto the x-direction actuator platform such that rotation of the stepper motor moves the y-direction platform up and down relative to the optical table surface. Identical motor drivers control each of the stepper motors. An Arduino interfaces with both drivers, enabling synchronous motion of the stage in the x and y directions. During the experiment, the stage shifts to expose each sample to the laser in turn, then holds so that spectral PL data can be acquired. The hold time is 15 seconds per sample, and data is collected from each sample every 6 minutes. The laser moves to a "rest position" after each sample, avoiding continuous light exposure. All samples are simultaneously exposed to the same environmental conditions and experience identical laser excitation for the same total amount of time.

III. Relative humidity control

The experimental setup, shown in Figure S1, consists of several parts that work in tandem to meet the design objectives. A custom enclosure houses multiple samples, and a translation stage allows all samples to be measured within a single experiment. These systems are integrated into a bulk PL setup consisting of an excitation laser (532 nm), various filters and collection optics, and a spectrometer to record the resulting spectra. Relative humidity is controlled using dry and wet N_2 lines. The wet line flow passes through a water bubbler to introduce moisture, and the rate of flow is adjusted using a mass flow controller (MFC) to ensure precise control and reproducibility. A temperature and humidity sensor with a ±1.8% relative humidity accuracy and a ±0.2◦C temperature accuracy is mounted in the chamber. All samples are located within 10 cm of the sensor. Sensor values are logged by an Arduino at regular intervals coinciding with acquisition of PL spectra.

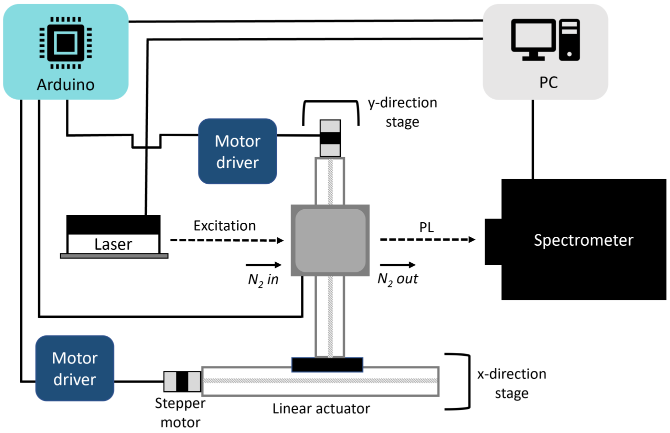

Figure S1: Schematic of automated PL setup. Python code on a PC coordinates the laser excitation source, spectrometer, and Arduino. Motion of the translation stage is controlled by two motor driver-stepper motor-linear actuator assemblies. The Arduino records environmental data from a sensor inside the sample chamber and operates both motor drivers. Inert N_2 gas is flowed through the chamber for the duration of the experiment.

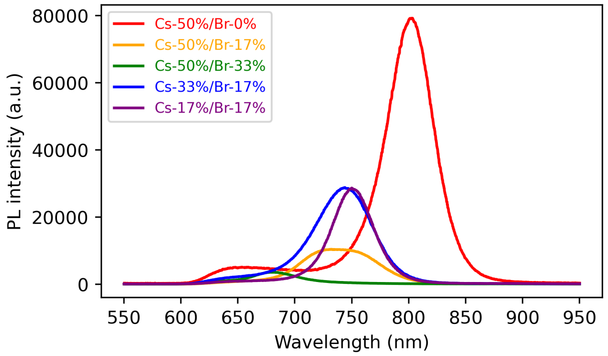

Figure S2: PL spectra of all samples prior to any exposure to high humidity levels.

III. Machine learning analysis

A. Data processing

Our data is acquired in the form of PL spectra at sequential time points. The temperature and rH in the chamber are measured simultaneously and their values are associated with each spectrum. Sampling rates for all experiments are adjusted so that every sample is measured at 6-minute intervals, for a total of 10 spectra per hour per sample. With five samples, this amounts to 50 spectra per hour and 7,200 total spectra for our 144-hour experiments.

Data is sorted into a dictionary with keys for each sample ID, based on experimental order. The PL .csv files are divided by sample and inserted into the dictionary as wavelength vs intensity. Calculations are performed on each file to extract the maximum PL value (a.u.), the peak location (in nm), the integrated absolute PL (a.u.), and the FWHM (in nm). In summary, each "data point" contains the following information:

(1) Full PL spectral data as wavelength vs intensity

(2) Time at which spectrum was acquired (in minutes, from beginning of the experiment)

(3) Temperature and rH at the time the spectrum was acquired

(4) Spectrum figures of merit (maximum PL, peak location, absolute PL, FWHM)

To prepare the data for ML, we first trim the dataset so that only the hours of rH cycling are included. In other words, we remove hours at the beginning and end of the dataset where the samples were held in steady environments to equilibrate. We are left with 118 hours of data for the rH cycling (Figure 3a). This is important because our time series forecasting models will learn long-term trends which imitate real-world weather conditions and segments where the environment is artificially held constant will negatively impact learning and predictive capability. Next, we normalize the rH and maximum PL such that the initial PL spectra maximum is equal to 1 for each sample, and the rH values are normalized from 0 to 1. This is necessary for the neural network activation functions to avoid runaway growth of network weights. [[3]]

B. Linear regression predictions on environmental PL data

We apply a baseline linear regression model to the PL time series, where temperature (T) and rH (Figure 4a) are the sole inputs to the regression. The model is trained on 50% of the data using a least-squares curve fitting method to determine the a, b, and c constants. The remaining 50% of the data is used for testing. The regression equation to predict the PL at time n is as follows:

$$PL_{pred}(n) = a * T(n) + b * rH(n) + c$$

The test performance is evaluated using the normalized root mean square error (NRMSE):

$$NRMSE(\%) = 100 * \frac{\sqrt{\sum_{i=1}^{n} \frac{(\hat{y}_i - y_i)^2}{n}}}{y_{max} - y_{min}}$$

Where n is the number of data points, $\hat{y}$ is the predicted PL, and y is the experimentally observed PL. The normalization step ($y_{max} - y_{min}$) enables direct quantitative comparison between all ML models.

C. Echo state network predictions on environmental PL data

An Echo State Network (ESN) is applied to the environmental PL data. [4] This type of recurrent neural network (RNN) is uniquely suited to time series analysis and allows them to learn historical trends in the data. ESN have a sparsely connected hidden layer, also called a "reservoir," in which not all neurons are linked to one another. This mitigates the vanishing gradient problem, where small values propagated through neural layers in a deep network go to zero as they are repeatedly multiplied. Because the networks learn through gradient descent and requires nonzero gradients for effective predictions, we use sparse connections as in ESN so that small values are not self-multiplied as often and better retained in the network. The structure of ESN also reduces the time required to train the network when compared to other RNN variants.

We use an open-source Python implementation (pyESN) as our ESN algorithm. The network contains 250 nodes with a sparsity of 0.1. We use 50% of the data for training and validation and 50% for testing. During training, the ESN updates the network states in a sparsely connected neural network to minimize the NRMSE. The update equation for each network state (X) is:

$$X(n) = tanh(W_{in}[rH, T(n)] + WX(n-1)) + Cv(n)$$

The prediction function is:

$$PL_{pred}(n) = W_{out}[rH, T(n) : X(n)]$$

The hyperbolic tangent is used as the activation function for network updates. The network weights – input weight matrix W_{in}, output weight matrix W_{out}, and reservoir weight matrix W – are randomly generated at the start of the training. The update process uses the input weights for the incoming environmental data vector rH, $T(n)$ and the reservoir weights from he previous time step $X(n-1)$. Random noise is added as a regularization parameter to mitigate overfitting, where C is the noise scaling and $v(n)$ is the noise vector. The prediction function uses the output weight matrix, the environmental data vector, and the network states set during training to calculate the PL at time

n. Network hyperparameters (noise and spectral radius) are set using a grid search approach on the validation set. The predictive performance is then evaluated using the test set.

The network has two additional hyperparameters which must be tuned prior to model evaluation. These are noise, a regularization hyperparameter that adds random noise to each neuron, and spectral radius of the recurrent weight matrix, which is a scaling parameter for the matrix eigenvalues. We use a grid search approach to optimize these values for each sample (see Figure S3). For this process, we subdivide the non-test data into training and validation sets, with a 25%-25%-50% train-validation-test split. This is a testing-heavy split which we select to probe the limits of ESN in long-term prediction tasks, as required to forecast the possible degradation of halide perovskites upon exposure to moisture.

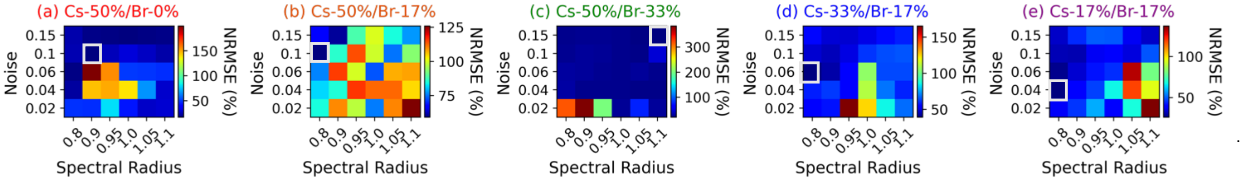

Figure S3: Optimizing ESN hyperparameters for rH-dependent PL with a grid search approach. Heatmaps for samples (a) Cs-50%/Br-0%, (b) Cs-50%/Br-17%, (c) Cs-50%/Br-33%, (d) Cs-33%/Br-17%, and (e) Cs-17%/Br-17%. The network is trained on each combination of hyper-parameters (noise and spectral radius) and the NRMSE is calculated from validation data predictions. The white boxes indicate the hyperparameters selected for each model.

D. Auto-regressive integrated moving average predictions on environmental PL data

We generate statistical time series forecasting models for the PL data. An Auto-Regressive Integrated Moving Average with eXogenous regressors (ARIMAX) model is used as the baseline for the rH cycling data. We also introduce a seasonality component to the algorithm and use a Seasonal ARIMAX (or SARIMAX) model. Again, 50% of the data is used for training and 50% for testing. The (p,d,q) parameters for ARIMAX and the additional (P,D,Q,s) parameters for SARIMAX are determined using stationarity tests and examination of the autocorrelation and partial autocorrelation plots for the training data. The full SARIMAX equation is [5]:

$$\Delta^d \Delta_s^D PL_{t,pred} = \theta(B)^p \Delta^d PL_t + \phi(B)^q \Delta^d \epsilon_t + \beta_{rH} rH_t + \beta_T T_t + \Theta(B)^P \Delta_s^D PL_t + \Phi(B)^Q \Delta_s^D \epsilon_t + \Delta^d \Delta_s^D \epsilon_t$$

Where Δ is the differencing operator and B is the backshift operator. Therefore, the $\theta(B)^p\Delta^d PL_t$ term represents a polynomial function with terms for each lagged time step up to p for the differenced time series. The coefficients for each term are included in θ and d is the order of differencing applied to the data. The other terms in the equation are interpreted as follows:

• $\Delta^d\Delta^D{}_S\, PL_{t,pred}$: predicts PL value at time t with differencing of order d and seasonal differencing of order D applied

• $\varphi(B\,)^q\ \Delta^d\ \varepsilon_t$: polynomial function of the lagged error terms with order q, coefficients φ, and differencing of order d applied

• $\beta_{rH} rH_t$: rH value at time t multiplied by coefficient β_{rH}

•βTTt: temperature value at time t multiplied by coefficient β_T

• $\Theta(B)^P\Delta^D{}_S\, PLt$: polynomial function of lagged seasonal terms with order P, coefficients Θ, and differencing order of D applied

• $\Phi(B)^Q\Delta^D{}_S\ \varepsilon t$: polynomial function of the lagged seasonal error terms with order Q, coefficients Φ, and differencing order of D applied

• $\Delta^d\ \Delta^D{}_S\ \varepsilon_t$: error term with differencing of order d and seasonal differencing of order D

For ARIMAX, the auto-regressive terms (p) are added to incorporate the effects of past PL measurements on the PL at the current time. For example, p=1 adds a term for the PL value at $t-1$ while p=2 adds terms for the PL values at $t-1$ and $t-2$. The integrative parameter (d) corresponds to the order of differencing, where a differenced time series is simply equal to the change between points in the original time series. Moving average terms (q) are like auto-regressive terms but relate to the error at past time steps rather than the PL value. Exogenous variables provide additional input to the model and in this case are rH and temperature.

Prior to fitting, the (p,d,q) of the ARIMAX model must be set. One common way of determining these values is by deconstructing the time series and visualizing lags between adjacent time steps.[6] Figures S4 and S5 show the plots used to choose an appropriate (p,d,q). For ARIMAX, the terms including seasonality (5th and 6th) and all seasonal differencing operators

$\Delta^D{}_S$ are removed. During training, all coefficients (θ, φ, β_{rH}, β_T, Θ, Φ) are set. The predictive performance is then evaluated using the test set.

To maintain a composition-agnostic framework as in the LR and ESN implementations, we deconstruct only the time series for a single sample and use the results to extrapolate to other compositions. Sample Cs-50%/Br-17% is selected for deconstruction as it has intermediate Cs:FA and Br:I ratios, with a full chemical formula of $(Cs_{3/6}FA_{3/6})Pb(Br_{1/2}I_{5/2})$. First, the latter 50% of the data is set aside for testing. The training data shows both a long-term downward trend and a strong seasonality (Figure S4a). This result is evidence of non-stationarity in the time series, meaning that the mean and variance of the data change over time. For SARIMAX to be effective, we take the first order difference and the seasonal difference (Figure S4b) to obtain a stationary data set. The SARIMAX hyperparameters are (P,D,Q,s) where s is the season length – in this case 60 data points (i.e. one 6-hour cycle). (P,D,Q) are equivalent to the ARIMAX (p,d,q) but on the seasonal level, meaning that they are relative to the PL at 60 lags. Taking the first difference of the Sample Cs-50%/Br-17% data and applying seasonal differencing (Figure S4) effectively creates a stationary time series.

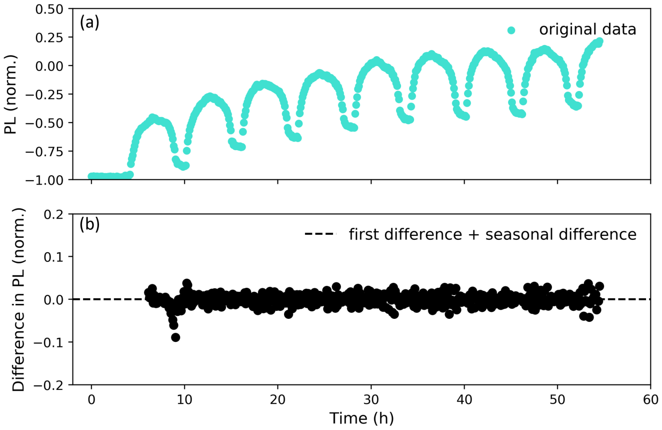

Figure S4: Original and twice-differenced rH-dependent PL time series for Cs-50%-Br-17%. (a) Training data after preprocessing. Values are normalized between -1 and 1. A 50-50% train-test split is applied. (b) Differenced training data. Two differencing steps are applied. (1) First order differencing: the PL value of the immediately preceding time step is subtracted from each point. The first data point is removed because it has no preceding time step. (2) First order seasonal differencing: the PL value of the time step at lag 60 is subtracted from each point. Lag 60 is selected to match the number of data points in each 6-hour rH cycle. The first 60 data points are removed.

Figure S5 shows the autocorrelation and partial autocorrelation for the differenced data. The twice differenced rH cycling data shows negative autocorrelation and partial autocorrelation at lag 1. This is an indication that moving average terms should be introduced. [7] The final SARIMAX selected is (0,1,1) (0,1,1,60). The differencing on the deconstructed time series for the Cs-50%/Br-17% sample shows that the first difference and first seasonal difference are required to produce stationarity. Therefore, we set both *d* and *D* equal to 1 for samples Cs-50%/Br-17%, Cs-33%/Br-17%, and Cs-17%/Br-17%. For samples that do not show non-seasonal trends (Cs-50%/Br-0% and Cs-50%/Br-33%) in their training data, we set $d = 0$ and $D = 1$. The seasonality components increase the computational complexity, and the time to fit the model increases to 60-120 seconds for a 16 GB RAM computer. Although significantly greater than the fitting time for the other ML models, this time is still several orders of magnitude lower than the SARIMAX forecasting window (50+ hours). We also caution that exact fitting times depend heavily on the specifications of the PC and how many background processes are running simultaneously. However, the order of magnitude fit time should be consistent across similar devices. The SARIMAX results show excellent visual fit (Figure 4k-o) and the average NRMSE is only 7.7%.

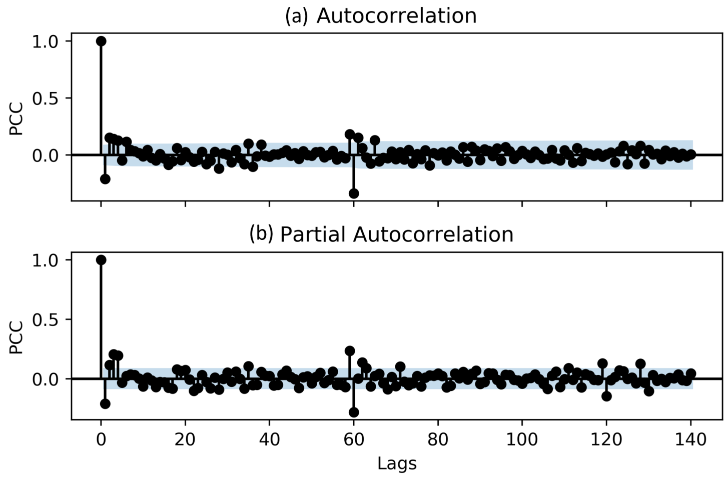

Figure S5: Autocorrelation and partial autocorrelation for the twice-differenced rH-dependent PL time series. (a) Pearson correlation coefficient (PCC) between a time point (lag 0) and the 140 preceding time steps in Cs-50%/Br-17% differenced training data. Includes both direct and indirect correlations. (b) Direct correlations between a time point and the 140 preceding time steps, with indirect correlations removed. The light blue area denotes the 95% confidence interval. Correlations within this interval can be attributed to random fluctuations.

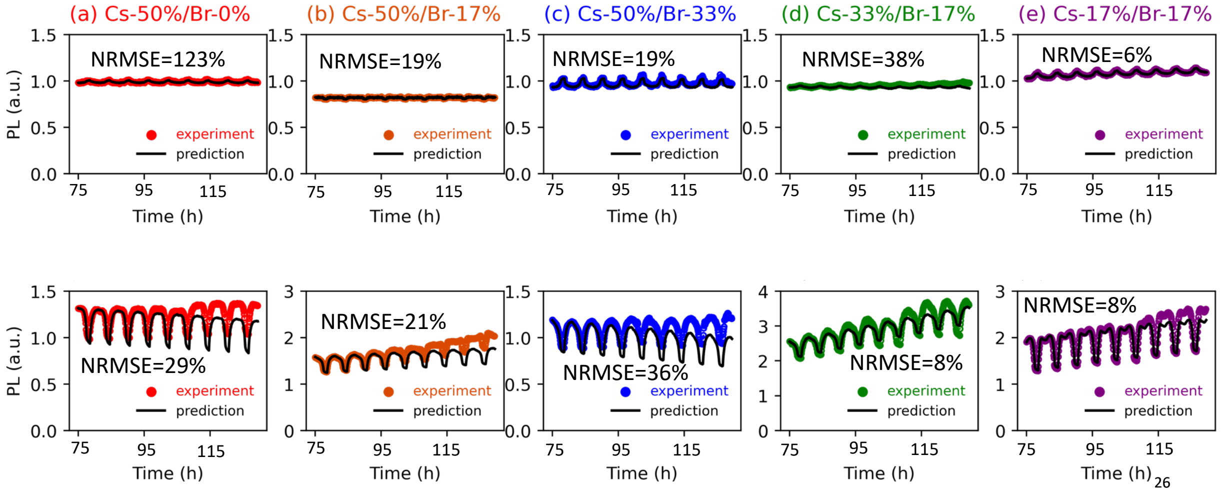

Figure S6: SARIMAX model applied to (top row) the FWHM and (bottom row) the integrated PL signal for each hybrid perovskite sample. A multi-Gaussian fit is used to analyze the evolution of the PL intensity and FWHM of side peaks. All NRMSE are displayed as insets. The integrated PL results account the area under all peaks, which may explain why the multipeak samples (such as a and c) have higher NRMSE.

References:

(1) An, Y.; Perini, C. A. R.; Hidalgo, J.; Castro-Méndez, A.-F.; Vagott, J. N.; Li, R.; Saidi, W. A.; Wang, S.; Li, X.; Correa-Baena, J.-P. Identifying High-Performance and Durable Methylammonium-Free Lead Halide Perovskites via High-Throughput Synthesis and Characterization. *Energy Environ. Sci.* **2021**, *14* (12), 6638–6654. https://doi.org/10.1039/D1EE02691G.

(2) Kirchartz, T.; Márquez, J. A.; Stolterfoht, M.; Unold, T. Photoluminescence-Based Characterization of Halide Perovskites for Photovoltaics. *Adv. Energy Mater.* **2020**, *10* (26), 1904134. https://doi.org/10.1002/aenm.201904134.

(3) Saidi, W. A.; Shadid, W.; Castelli, I. E. Machine-Learning Structural and Electronic Properties of Metal Halide Perovskites Using a Hierarchical Convolutional Neural Network. *Npj Comput. Mater.* **2020**, *6* (1), 36. https://doi.org/10.1038/s41524-020-0307-8.

(4) Jaeger, H.; Haas, H. Harnessing Nonlinearity: Predicting Chaotic Systems and Saving Energy in Wireless Communication. *Science* **2004**, *304* (5667), 78–80. https://doi.org/10.1126/science.1091277.

(5) Newbold, P. ARIMA Model Building and the Time Series Analysis Approach to Forecasting. *J. Forecast.* **1983**, *2* (1), 23–35. https://doi.org/10.1002/for.3980020104.

(6) J. Contreras; R. Espinola; F. J. Nogales; A. J. Conejo. ARIMA Models to Predict Next-Day Electricity Prices. *IEEE Trans. Power Syst.* **2003**, *18* (3), 1014–1020. https://doi.org/10.1109/TPWRS.2002.804943.

(7) Tunnicliffe Wilson, G. Time Series Analysis: Forecasting and Control,5th Edition, by George E. P. Box, Gwilym M. Jenkins, Gregory C. Reinsel and Greta M. Ljung, 2015. Published by John Wiley and Sons Inc., Hoboken, New Jersey, Pp. 712. ISBN: 978-1-118-67502-1. *J. Time Ser. Anal.* **2016**, *37*, n/a-n/a. https://doi.org/10.1111/jtsa.12194.
